# Supplementary material for: A Case of In Situ Phage Therapy against Staphylococcus aureus in a Bone Allograft Polymicrobial Biofilm Infection: Outcomes and Phage-Antibiotic Interactions
Source: Viruses. 2021 Sep 22;13(10):1898. doi: 10.3390/v13101898 (PMC8539586; doi:10.3390/v13101898)
Supplement: Supplementary file 1 [file viruses-13-01898-s001.zip › viruses-1349613-supplementary.pdf]

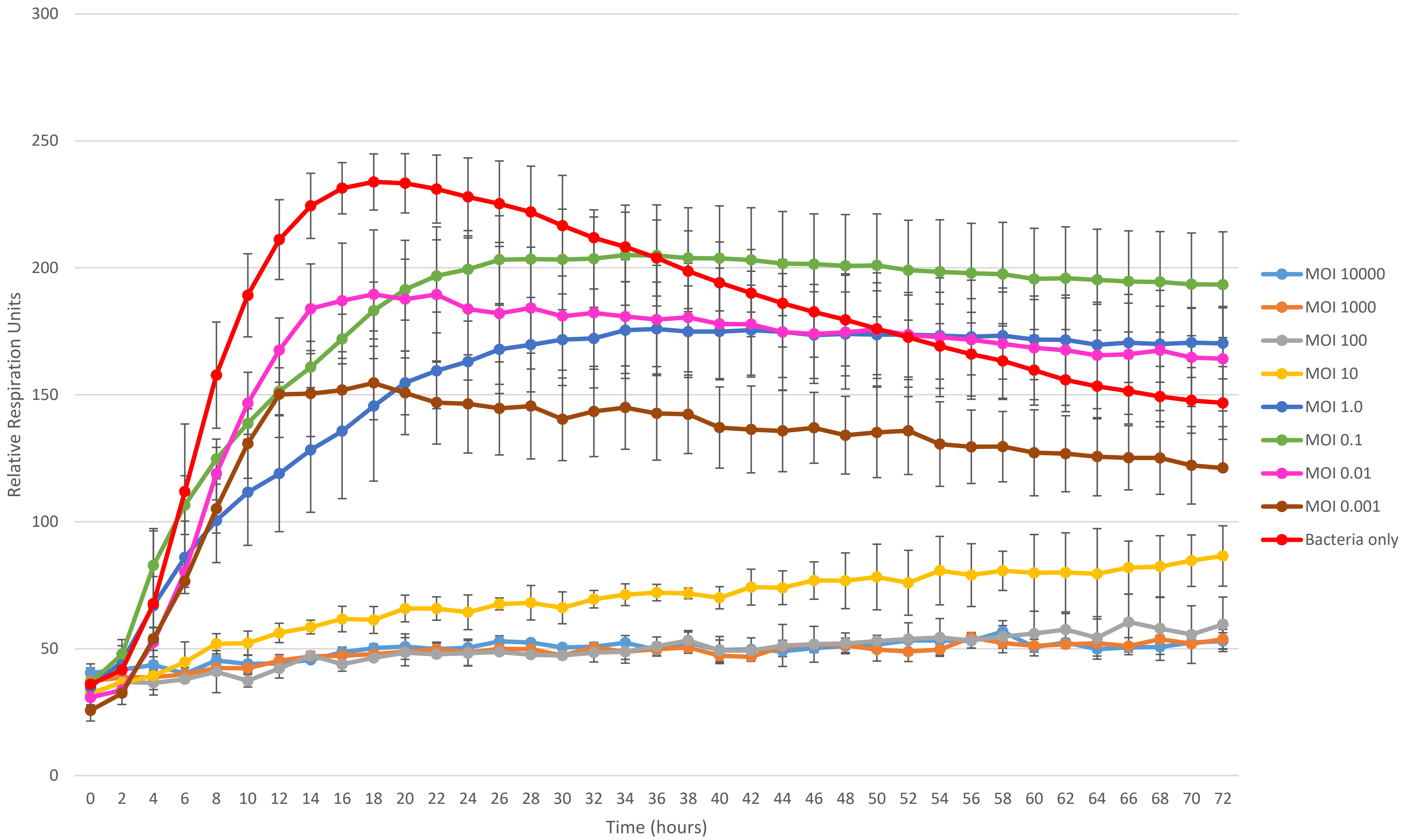

Figure S1. Assessment of bacterial growth kinetics with the OmniLog® system. *S. aureus* strain with phage ISP at different Multiplicities Of Infection (MOI).

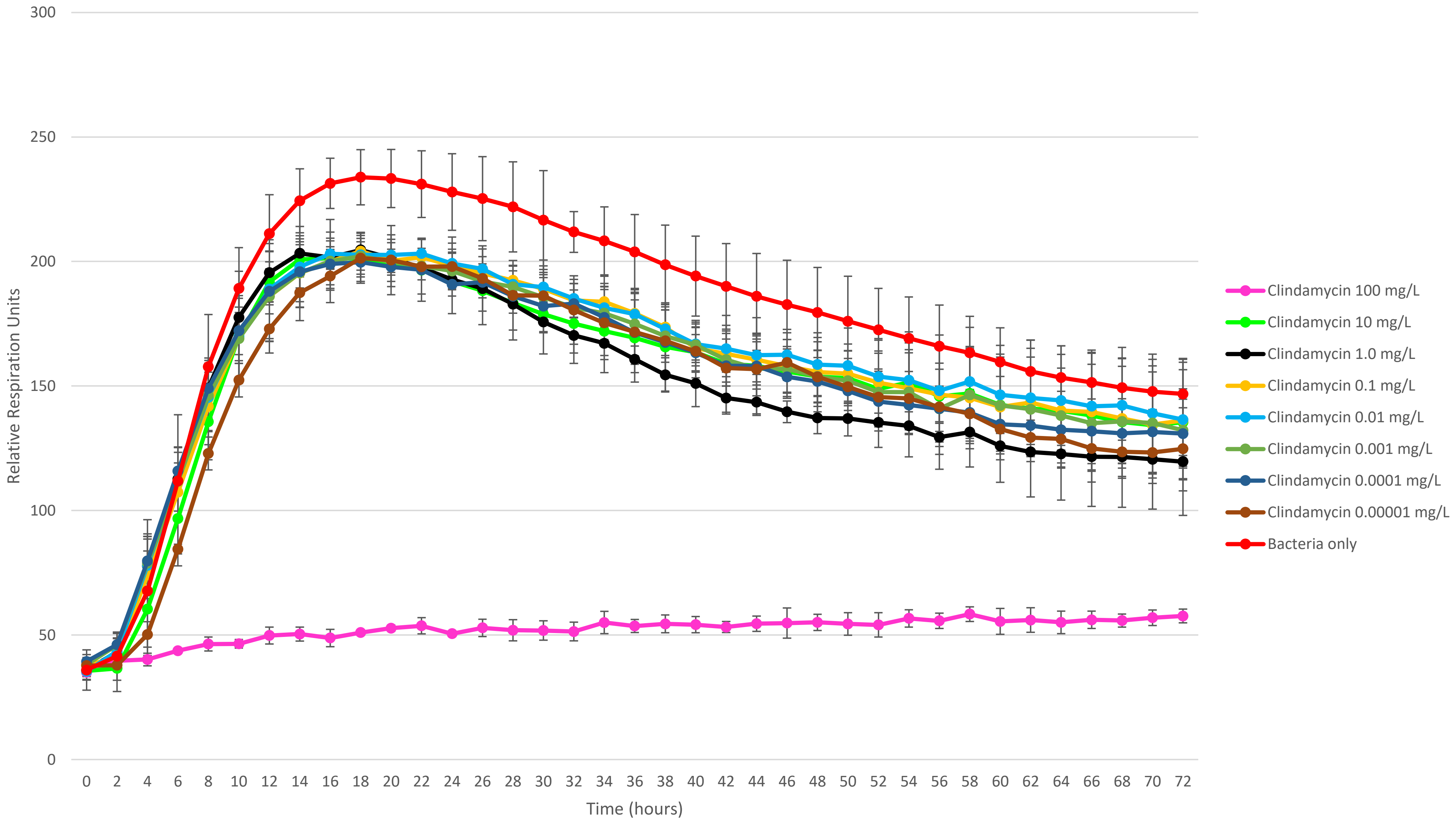

Figure S2. Assessment of bacterial growth kinetics with the OmniLog® system. *S. aureus* strain with clindamycin at different concentrations.

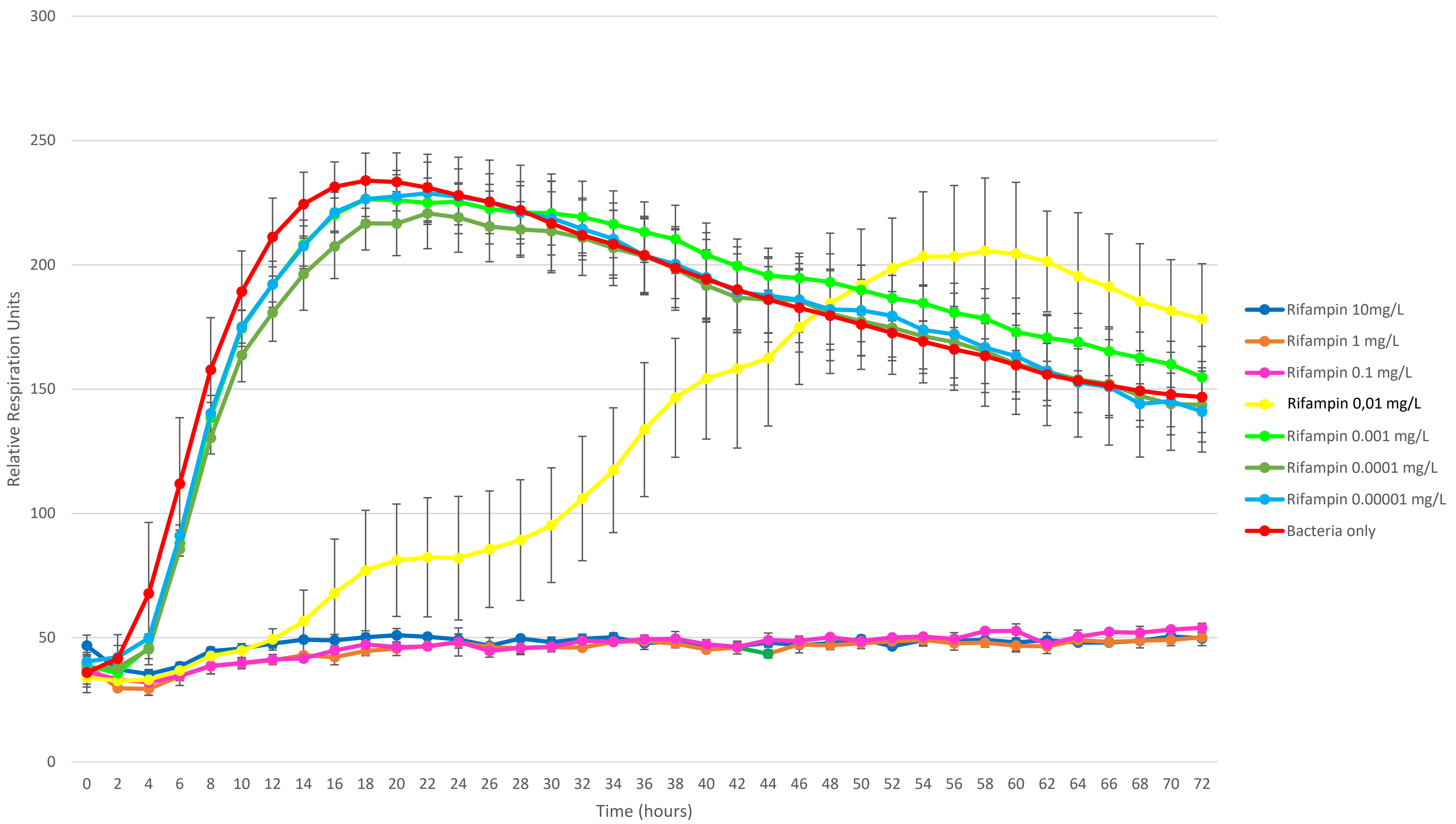

Figure S3. Assessment of bacterial growth kinetics with the OmniLog® system. *S. aureus* strain with rifampin at different concentrations.

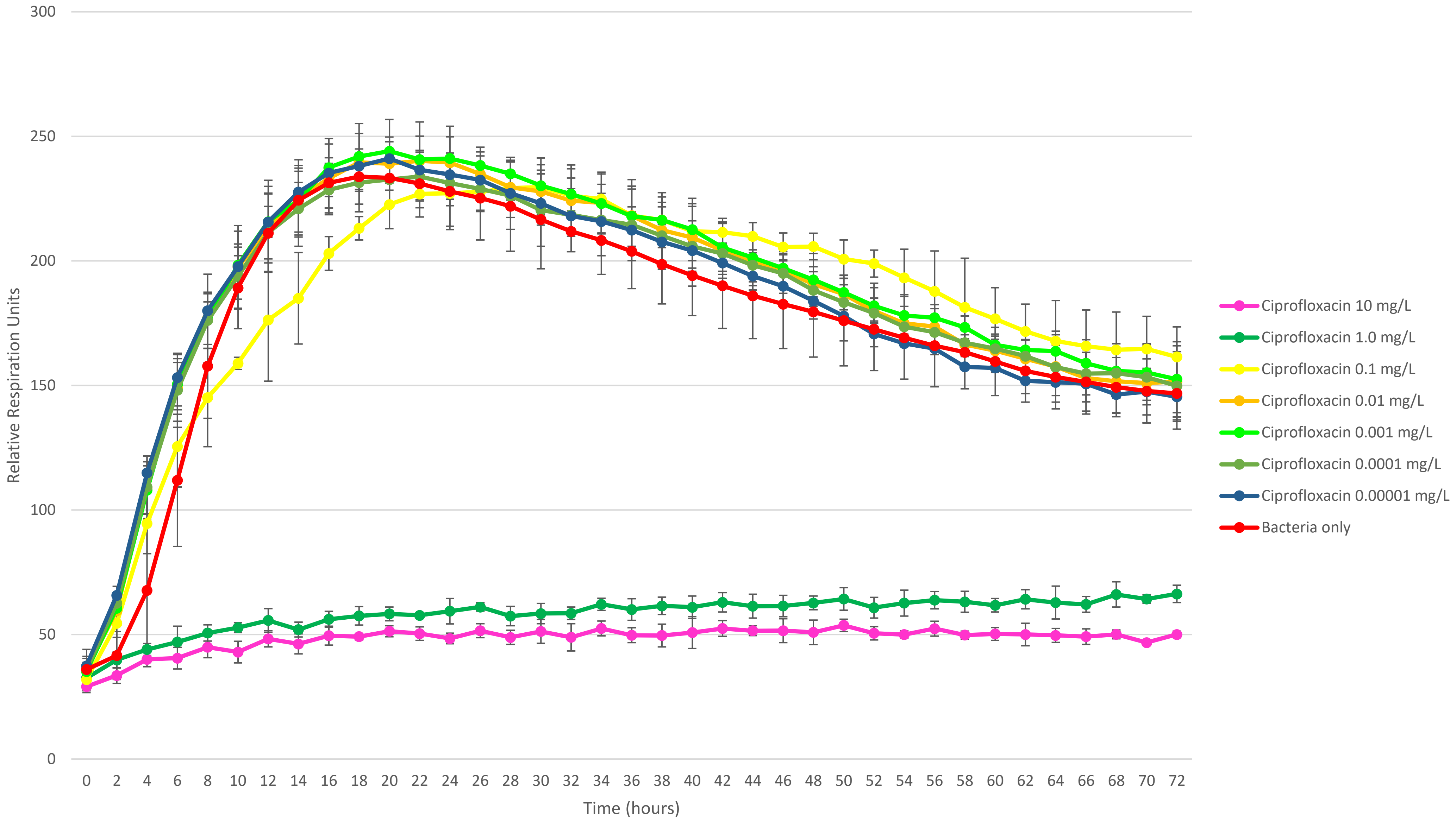

Figure S4. Assessment of bacterial growth kinetics with the OmniLog® system. *S. aureus* strain with ciprofloxacin at different concentrations.

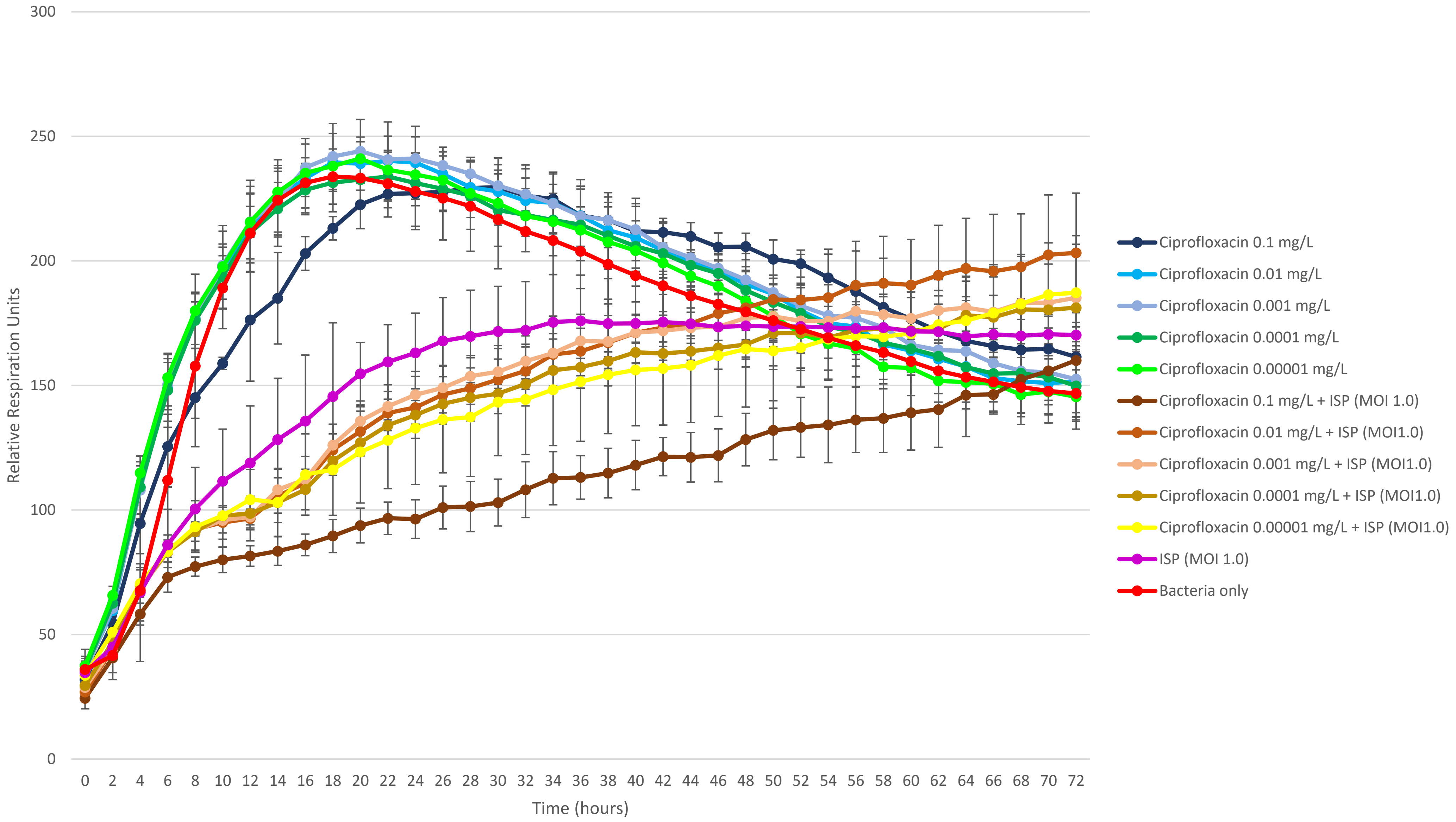

Figure S5. Assessment of bacterial growth kinetics with the OmniLog® system. *S. aureus* strain with ciprofloxacin at different concentrations and phage ISP at MOI = 1,0.

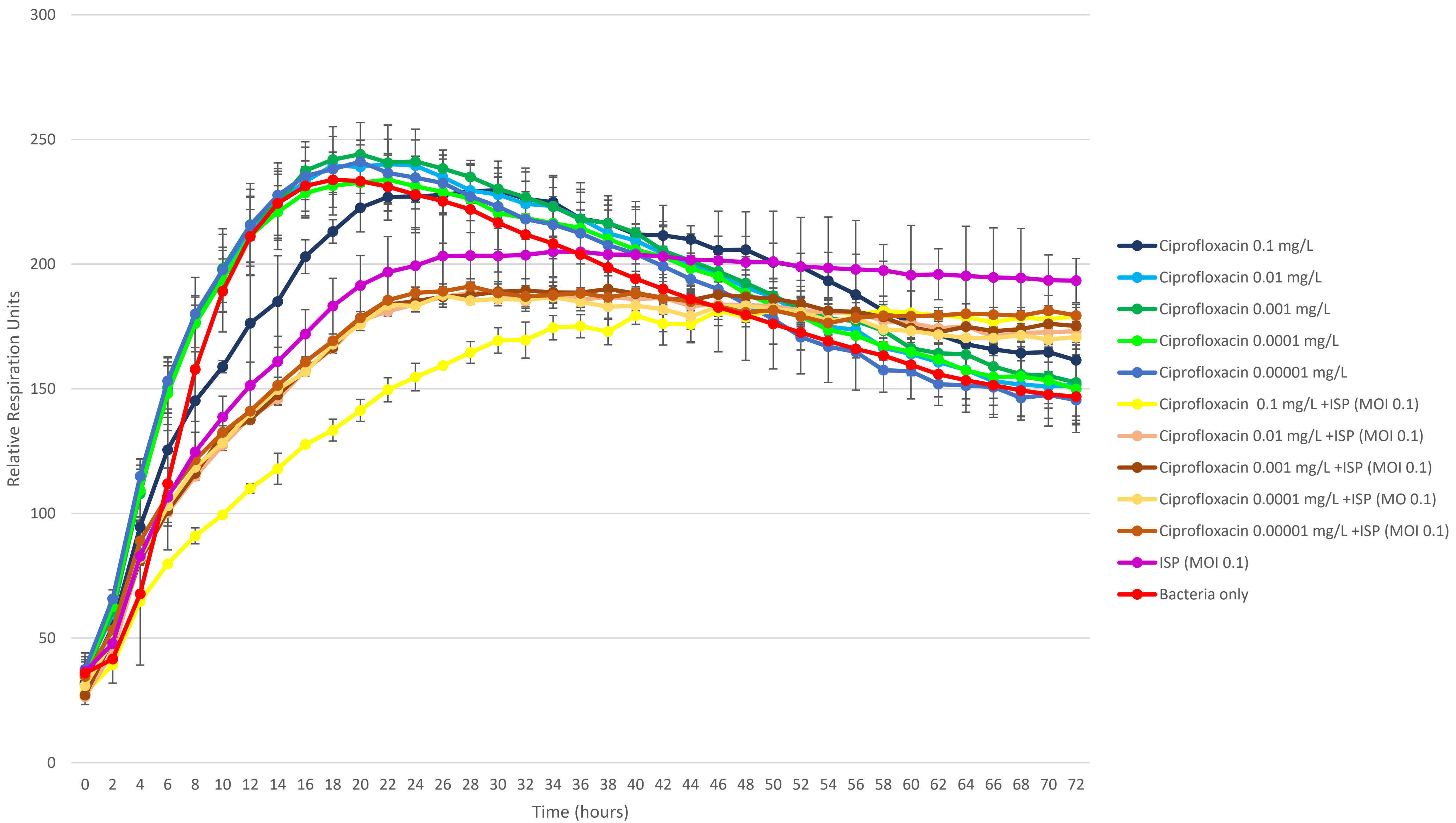

Figure S6. Assessment of bacterial growth kinetics with the OmniLog® system.
S. aureus strain with ciprofloxacin at different concentrations and phage ISP at MOI = 0,1.
